# In Vitro Assessment of Nanosilver-Functionalized PMMA Bone Cement on Primary Human Mesenchymal Stem Cells and Osteoblasts

**DOI:** 10.1371/journal.pone.0114740

**Published:** 2014-12-08

**Authors:** Linda Pauksch, Sonja Hartmann, Gabor Szalay, Volker Alt, Katrin S. Lips

**Affiliations:** 1 Laboratory for Experimental Trauma Surgery, Justus-Liebig University Giessen, Giessen, Germany; 2 Department of Trauma Surgery, University Hospital Giessen and Marburg, Giessen, Germany; University of Sheffield, United Kingdom

## Abstract

Peri-prosthetic infections caused by multidrug resistant bacteria have become a serious problem in surgery and orthopedics. The aim is to introduce biomaterials that avoid implant-related infections caused by multiresistant bacteria. The efficacy of silver nanoparticles (AgNP) against a broad spectrum of bacteria and against multiresistant pathogens has been repeatedly described. In the present study polymethylmethacrylate (PMMA) bone cement functionalized with AgNP and/or gentamicin were tested regarding their biocompatibility with bone forming cells. Therefore, influences on viability, cell number and differentiation of primary human mesenchymal stem cells (MSCs) and MSCs cultured in osteogenic differentiation media (MSC-OM) caused by the implant materials were studied. Furthermore, the growth behavior and the morphology of the cells on the testing material were observed. Finally, we examined the induction of cell stress, regarding antioxidative defense and endoplasmatic reticulum stress. We demonstrated similar cytocompatibility of PMMA loaded with AgNP compared to plain PMMA or PMMA loaded with gentamicin. There was no decrease in cell number, viability and osteogenic differentiation and no induction of cell stress for all three PMMA variants after 21 days. Addition of gentamicin to AgNP-loaded PMMA led to a slight decrease in osteogenic differentiation. Also an increase in cell stress was detectable for PMMA loaded with gentamicin and AgNP. In conclusion, supplementation of PMMA bone cement with gentamicin, AgNP, and both results in bone implants with an antibacterial potency and suitable cytocompatibility in MSCs and MSC-OM.

## Introduction

Due to its low cost, excellent biocompatibility and mechanical strength polymethylmethacrylate (PMMA) bone cement is successfully used in fixation of artificial joints and as a filling material for bone cavities. But PMMA bone cement, like other biomaterials, bears the risk of microbial colonization. The incidence of peri-prosthetic infections ranges between 0.5–5% [1]–[5] and is further elevated for revisions [6]–[8]. In the case of PMMA the rates of infection can even increase to 13% [9]. Peri-prosthetic infections often require the removal of the implant, which displays not only a devastating situation for the patient but also a high economic burden, with costs exceeding $50,000 [10], [11].

The interstitial milieu surrounding an implant is known as a region of depressed immune function [12]. This milieu promotes the settlement of pathogens on the implant surface and the formation of biofilms. The majority of implant-associated infections are caused by gram positive *staphylococci*, like *Staphylococcus aureus* or *Staphylococcus epidermidis* [13], [14]. To reduce the risk of bacterial colonization a systemic antibiosis is routinely applied. There have also been several promising approaches of loading PMMA bone cement with antibiotics, like gentamicin or vancomycin [15]–[17]. A distinction is made in using antibiotic-loaded PMMA for prophylactic therapy and for infection treatment. To counteract an infection, PMMA has to be loaded with concentrations ≥3.6 g antibiotic per 40 g PMMA powder. Low-dose antibiotic loaded PMMA, with concentrations ≤1 g, is routinely used in the prevention of bacteria settlement during the first days [15]. When considering the prophylactic use of antibiotic loaded PMMA, the incorporated antibiotic has to cover a broad spectrum including gram-positive and gram-negative bacteria.

However, in the last decades the rate of biomaterial-infections caused by multidrug resistant bacteria has drastically increased [1]. Therefore, an adequate prophylaxis against such organisms has to be established.

The excellent antimicrobial effect of silver nanoparticles (AgNP) and its ability to reduce biofilm formation has been demonstrated in several studies [18]–[23]. AgNP even hold the capacity of inhibiting multidrug resistant bacteria [24]–[26]. The oligodynamic effect of nanosilver depends on the steady release of silver ions. The positively charged ions preferably bind to electron donor groups like sulphur or phosphorus. Therefore, AgNP are able to interact with sulphur-containing metabolic enzymes of the electron transport chain or with the phosphorus-rich DNA, leading to inhibition of bacterial energy production and replication [27]. In addition, it is suggested that AgNP can disrupt bacterial membrane by direct contact or indirectly by generation of reactive oxygen species [20], [28], [29]. This versatile mechanism of bacterial inhibition displays broad-spectrum antibacterial capacity and ensures that silver-resistance cannot easily be gained by point mutation.

One essential prerequisite for a successful antimicrobial implant material is excellent biocompatibility in the appropriate tissue. Therefore, a study was conducted to evaluate the biocompatibility of PMMA bone cement functionalized with AgNP and/or gentamicin in primary human mesenchymal stem cells (MSCs) and MSCs cultured in osteogenic differentiation media (MSC-OM). Thereby, essential biological characteristics of the bone forming cells were evaluated in the presence of the different PMMA variants.

## Materials and Methods

### 2.1 Testing material

The PMMA bone cement samples with a diameter of 10 mm and a thickness of 3 mm were provided by aap Biomaterials GmbH (Dieburg, Germany). The PMMA bone cements were functionalized with 4000 µg/g AgNP (AgPURE W50, ras materials GmbH, Regensburg, Germany). Furthermore, some of the PMMA samples were additionally supplemented with 1.34% gentamicin sulfate. PMMA bone cement without any loading and PMMA bone cement solely supplemented with 1.34% gentamicin sulfate served as internal controls. The cement samples were mixed in a vacuum mixing system and afterwards filled into molds to receive cylindrical forms.

AgNP used in this study correspond to the OECD Representative Manufactured Nanomaterial NM-300 and characterization of the nanoparticles is given in our previous study [30], [31].

### 2.2 Cell culture

MSCs were isolated from human reaming debris with the permission of the local Ethics Commission of the Department of Medicine at the Justus-Liebig-University Giessen (249/12). Participants provided their written consent to participate in this study. The patients were of different gender and ages and did not display any diseases linked to the bone metabolism.

Briefly, tissue remaining at the drill head during intramedullary nailing was transferred to a petri dish with F12K medium including 20% FCS, 100 U/ml penicillin and 100 µg/g streptomycin. After 4–7 days MSCs started to grow out of the tissue. When a confluent monolayer was formed, the cells were trypsinized and transferred to cell culture flasks.

For induction of osteogenic differentiation the MSCs were kept in Dulbecco's modified Eagle's medium (DMEM) low glucose w/l-glutamine, 10% FCS, 100 U/ml penicillin, 100 µg/g streptomycin, 0.1 µM dexamethasone, 0.005 µM ascorbic acid and 10 mM β-glycerol phosphate and named MSCs in osteogenic media (MSC-OM).

All cells were incubated at 37°C and 5% CO_2_. The cell medium was changed every seventh day and the cells were no further passaged during the experiments.

### 2.3 Cell viability

To study cell viability of PMMA samples a MTT assay was conducted. 10,000 cells per cm^2^ were seeded as duplets into 24-well plates. Subsequently, test materials were added and one part of the cells was kept in MSC medium, while the other part was incubated with osteogenic differentiation medium. Cells without PMMA bone cement served as a negative control. At day 1, 7 and 21 after addition of test materials the MTT substance was given to the cell medium. After incubation in the dark for 4 hours at 37°C the cell medium was discarded and the cells were lyzed with 0.004 N HCl in isopropanol. After removal of the testing materials the lysates were centrifugated and the supernatants were transferred as triplets to a 96-well plate. Absorption was measured at 570 and 630 nm using the Synergy HT Microplate Reader (BioTek, Bad Friedrichshall, Germany).

### 2.4 Cell number determined by DNA content

To determine changes in the cell number a PicoGreen dsDNA quantitation assay (Invitrogen, Eugene, Oregon, USA) was performed. Prior to exposure to the testing materials, 10,000 cells per cm^2^ were seeded as duplets into 24-well plates. Subsequently, the medium was changed to differentiation medium and the PMMA samples were given to the cells. Cells without testing material served as a negative control. At day 1, 7 and 21 after addition of material cells were lyzed with 1% Triton X-100 in phosphate buffered saline, before removing the testing material. The lysates were frozen at −80°C, thawed and centrifugated. The supernatants were transferred as triplets to a 96-well plate and mixed with the PicoGreen working solution. The samples were excited at 485 nm and the fluorescence emission intensity was measured at 528 nm using the Synergy HT Microplate Reader.

### 2.5 Cell differentiation

Influences on osteogenic differentiation capacity were observed by performing a SensoLyte pNPP Alkaline Phosphatase assay (AnaSpec, Fremont, CA, USA). Therefore, 10,000 cells per cm^2^ were seeded as duplets into 24-well plates. Subsequently the medium was changed to differentiation medium and the testing materials were given to the cells. Cells without PMMA bone cement served as a negative control. At day 1, 7 and 21 the cells were lyzed with 1% Triton X-100 in phosphate buffered saline and the PMMA specimens were removed. Subsequently, the cell lysates were frozen at −80°C. After thawing, the samples were centrifugated and the supernatants were transferred as triplets to a 96-well plate. The samples were mixed with alkaline phosphatase substrate and the absorbance was measured at 405 nm with the Synergy HT Microplate Reader.

### 2.6 Cell morphology

The morphological reaction of the cells to the testing material was observed by inverted light microscopy and fluorescence microscopy. Therefore, the cells were seeded on the PMMA variants and either incubated with MSC medium for 7 days or with differentiation medium for 21 days. To monitor the outer cellular morphology by inverted light microscopy a Leica microscope TYPE 090-135.002 (Leica Microsystems GmbH, Wetzlar, Germany) equipped with a Nikon DS-Fi1 digital camera (Nikon, Duesseldorf, Germany) was used.

For fluorescence microscopy the samples were washed and fixed for 10 min with 4% paraformaldehyde in phosphate buffered saline, pH 7.3. Thereupon, the samples were washed, permeabilized for 5 min with 0.1% Triton X-100 in phosphate buffered saline and washed again. To display the distribution of F-actin in the cells, the samples were incubated with 5 µg/ml phalloidin-FITC (Sigma-Aldrich Chemie GmbH, Steinheim, Germany) for 40 min in the dark. Subsequently, the cells were washed and the DNA was stained by incubation with 2 µg/ml DAPI (Roth, Karlsruhe, Germany) for 15 min in the dark. Finally, the samples were mounted on glass coverslips with ProLong Gold Antifade Reagent (Invitrogen) and kept at 4°C for further analysis with the confocal laser scanning microscope (TCS SP5, Leica Microsystems GmbH) and the inverted fluorescence microscope (Olympus IX81, Olympus Deutschland GmbH, Hamburg, Germany).

### 2.7 Real time reverse transcription polymerase chain reaction

To analyze the influence of the testing materials on the gene expression of the MSCs and MSC-OMs, 10,000 cells per cm^2^ were seeded as duplets into 24-well plates. Subsequently, PMMA bone cements were given to the cells and one part of the cells was kept in MSC medium, while the other part was incubated in differentiation medium. Cells without testing material served as a negative control. The total RNA was isolated after 24 hours, 7 days and 21 days with the RNeasy Mini Kit (Qiagen GmbH, Hilden, Germany). Thereafter, cDNA was synthesized from 1000 ng of the isolated RNA using the QuantiTect Reverse Transcription Kit (Qiagen GmbH).

With the QuantiFast SYBR Green PCR Kit (Qiagen GmbH) the real-time polymerase chain reaction (PCR) was performed. Primers were purchased from Eurofins MWG Operon (Ebersberg, Germany) (Table 1). The results were normalized to the reference gene beta-2-microglobulin (β2MG).

**Table 1:** Listing of primers used to examine the influence of the testing material on different cell markers.

Gene	Role	MSC	MSC-OM	Forward primer (5′–3′)	Reverse primer (3′–5′)
β2MG (NM_004048)	Reference	X	X	TCTCTCTTTCTGGCCTGGAG	CAACTTCAATGTCGGATGGA
CCND1 (NM_053056.2)	Proliferation	X		CAACAACTTCCTGTCCTACT	GGGTCCATGTTCTGCT
Cav-1 (NM_001753.4)	Caveolae-dependent Endocytosis	X	X	ACGTAGACTCGGAGGGACAT	CAGGTCGATCTCCTTGGTGT
PICALM (NM_001206946.1)	Clathrin-dependent Endocytosis	X	X	ATTTTCTACCCCTAGTTCTTC	AGTAGCAGAGAAAGGATCTC
Nqo1 (NM_000903.2)	Oxidative stress	X	X	AGCTGGAAGCCGCAGACCTTG	TGAACACTCGCTCAAACCAGCC
CHOP (NM_001195053.1)	ER stress	X	X	ACCCTGCTTCTCTGGCTTGGC	GGAGAGTGAGGCCTCTGGGAG
ALP (NM_000478)	Osteogenic Differentiation		X	GGACATGCAGTACGAGCTGA	CCACCAAATGTGAAGACGTG
RUNX2 (NM_001015051.3)	Osteogenic Differentiation		X	GGCCTTCAAGGTGGTAGCC	ATCGTTACCCGCCATGAC

β2MG  =  beta-2-microglobulin, CCND1  =  cyclin D1, Cav-1  =  caveolin1, PICALM  =  phosphatidylinositol binding clathrin assembly protein, Nqo1  =  NADPH quinone oxidoreductase 1, CHOP  =  C/EBP homologous protein, ALP  =  alkaline phosphatase, RUNX2  =  runt-related transcription factor 2.

### 2.8 Statistical analysis

All experiments were repeated five times. The results are presented as means ± SEM. The data were statistical analyzed by Kolmogorov-Smirnov-Test or non-parametric Kruskal-Wallis using SPSS software (Version 20.0, SPSS Inc., IBM-Corp., Armonk, NY, USA). Pairwise multiple comparisons were performed using the Least Significant difference test for *post hoc* analysis after testing the variance homogeneity with the Levene test. For MTT, PicoGreen, ALP Assay and ALP gene expression, the treatment groups were compared with day 1 of the control group without addition of PMMA bone cement. For MTT also the Jonckheere trend test was performed. Regarding PCR of MSC the treatment groups were compared within the different time points while MSC-OMs were compared with day 1 of the control group. The probability of type I error less than or equal 0.05 was considered to be statistically significant.

## Results

### 3.1 Cell viability

To assess cell viability in MSCs and MSC-OMs caused by the different PMMA bone cements, a MTT assay was conducted.

**Figure 1:**
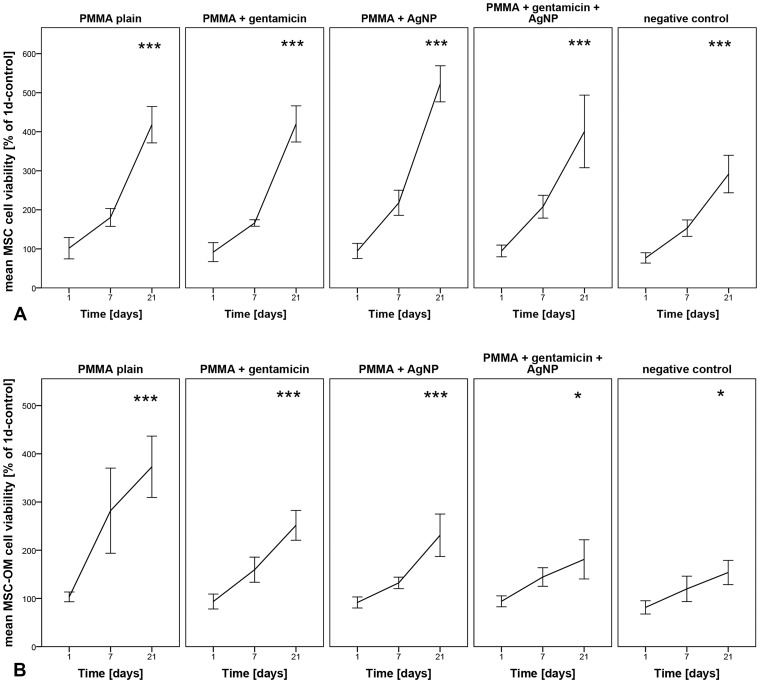
Cell viability determined by MTT assay. (A) Human mesenchymal stem cells (MSCs) and (B) MSCs cultured in osteogenic media (MSC-OM) were incubated with different PMMA bone cements for different time periods. Data are normalized to day 1 of controls and presented as mean ± SEM of five individual experiments (*p≤0.05, ***p≤0.001 vs. 1d-control w/o testing material).

In MSCs, no inhibition of cell viability, compared to the day 1 control without testing material, was detected at any time point. However, comparing the different materials PMMA loaded with AgNP revealed the highest level at day 21, and the negative control without testing material the lowest level in cell viability (Fig. 1A).

In MSC-OMs, plain PMMA showed the highest level of cell viability at day 21 and - similar to the MSCs - the negative control without testing material the lowest level in cell viability (Fig. 1B). No decrease in cell viability was detected from day 1 to 21. In comparison to the plain PMMA, decreased cell viability is seen for PMMA loaded with gentamicin, AgNP or gentamicin and AgNP (Fig. 1B).

Summarized, it becomes apparent that the cell viability of the different PMMA cements is at least similar as to the negative controls without material or increased.

### 3.2 Cell number & differentiation

**Figure 2:**
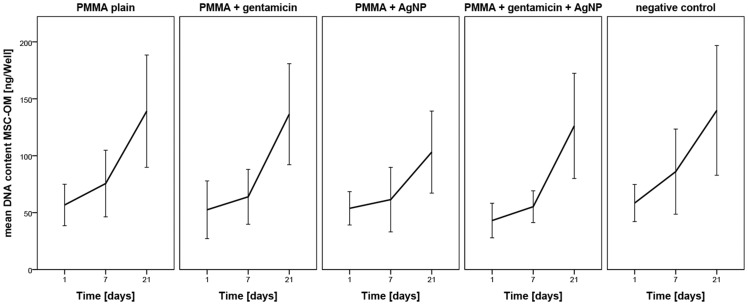
DNA content determined by PicoGreen assay. The effect of different PMMA bone cements on cell number and therefore proliferation of human mesenchymal stem cells cultured in osteogenic differentiation media at different time points normalized to day 1 of control cells without material. Data are presented as the mean ± SEM of five individual experiments.

A PicoGreen assay was performed to determine the cell number of MSCs cultured in osteogenic media. The values are normalized to day 1 of controls without material. From day 1 to day 21, there is a tendential increase in DNA content and therefore cell number for all tested PMMA samples similar as seen in the controls without application of material. No negative effects were detected for the different PMMA bone cements (Fig. 2).

**Figure 3:**
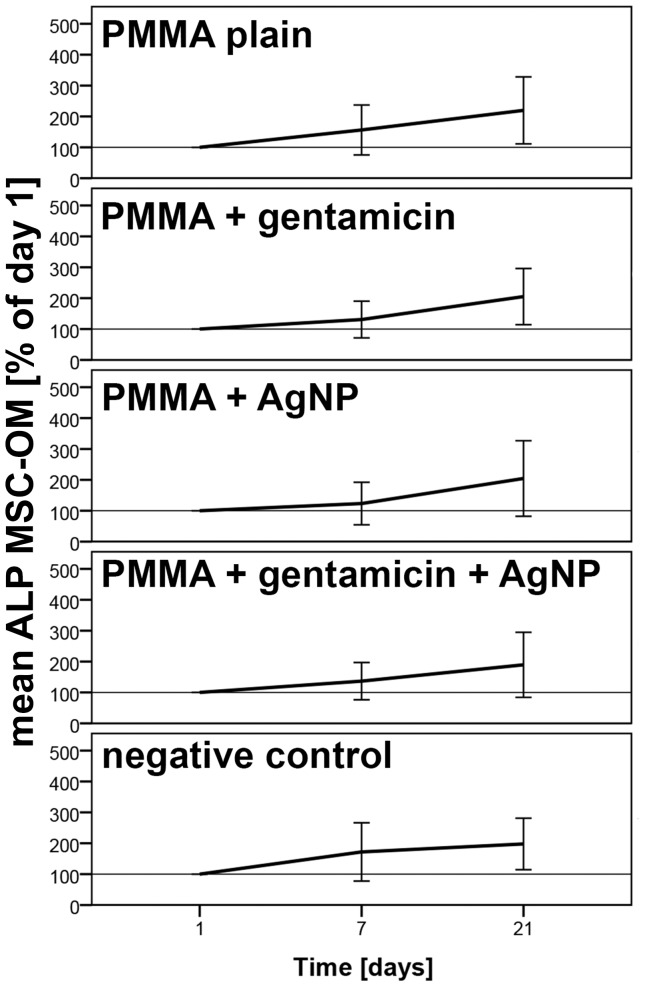
Alkaline Phosphatase amount determined by ALP assay. Influence of different PMMA bone cements on the differentiation behavior of mesenchymal stem cells cultured in osteogenic media (MSC-OM) at different time points normalized to day 1. Data are presented as the mean ± SEM of five individual experiments.

For determination of osteogenic differentiation the amount of alkaline phosphatase was measured using an alkaline phosphatase assay. No significant changes caused by the testing materials were observed. However, induction of MSC-OMs with the different PMMA bone cements did not decrease osteogenic differentiation capacity which was similar to the controls without addition of bone material (Fig. 3).

Summerized, it becomes apparent that PMMA loaded with AgNP and gentamicin does not decrease cellular proliferation and osteogenic differentiation capacity compared to plain PMMA and control cells without bone material. The lack of significant increase in cellular number and amount of alkaline phosphatase might be resulted by the considerable fluctuations in between the tested patients (Figs. 2 and 3).

### 3.3 Cell morphology

**Figure 4:**
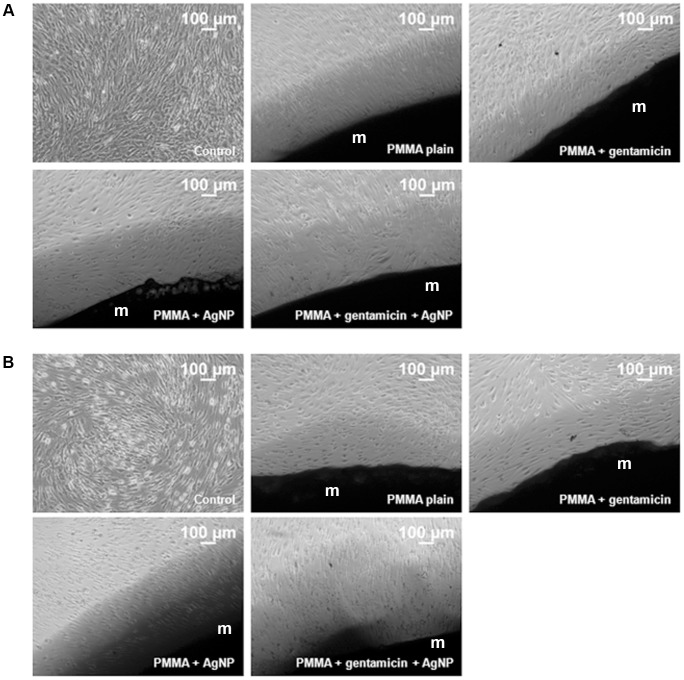
Microscopic observation of cellular morphology. (A) Human mesenchymal stem cells (MSCs) and (B) MSCs cultured in osteogenic differentiated media (MSC-OM) were incubated with different PMMA bone cements (m) for 21 days.

Cells incubated with the different PMMA variants for 21 days did not reveal significant changes in cell morphology according to inverted light microscopy. Like the control without testing material, the MSCs built up a confluent monolayer around the PMMA samples. All cells displayed a normal MSC shaped, elongated phenotype (Fig. 4A).

The MSC-OMs incubated with the testing material also built up a confluent monolayer, with healthy cells directly growing at the interface of monolayer and PMMA sample. The MSC-OMs of all PMMA groups displayed the normal contracted, oval morphology (Fig. 4B).

Fluorescence microscopy further revealed good attachment of MSCs and MSC-OMs to the surface of plain PMMA and PMMA with gentamicin and AgNP.

**Figure 5:**
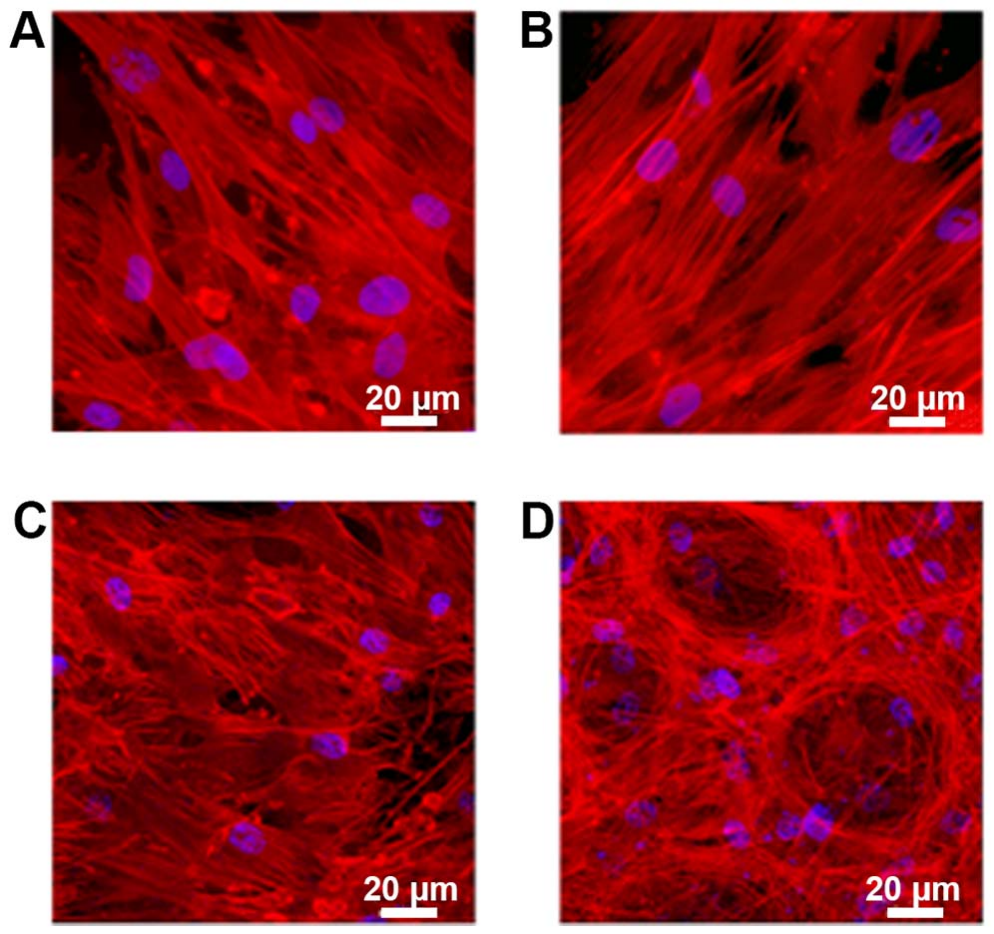
Cellular attachment observed by fluorescence microscopy. (A and B) human mesenchymal stem cells(MSCs) and (C and D) MSCs cultured in osteogenic differentiation media (MSC-OM) with (A and C) plain PMMA bone cement and (B and D) PMMA bone cement loaded with gentamicin and AgNP incubated for 7 days (MSC) and 21 days (MSC-OM), respectively.

MSCs incubated for 7 days on both PMMA types displayed an unchanged, elongated morphology. There are no visible abnormalities in cytoskeleton structure (Fig. 5A and B).

Also for the MSC-OMs incubated for 21 days on the plain PMMA bone cement or on PMMA supplemented with gentamicin and AgNP an undestroyed cytoskeleton structure could be confirmed.

Interestingly, the cells incubated on PMMA loaded with gentamicin and AgNP arranged in nest-like structures consisting of not fully differentiated cells. The MSC-OMs incubated on plain PMMA showed a very distinctive MSC-OMs phenotype, which indicates osteogenic differentiation (Fig. 5C and D).

### 3.4 Real time RT-PCR

To evaluate influences on the mRNA level caused by the testing materials, real time RT-PCR was conducted for specific cell markers.

**Figure 6:**
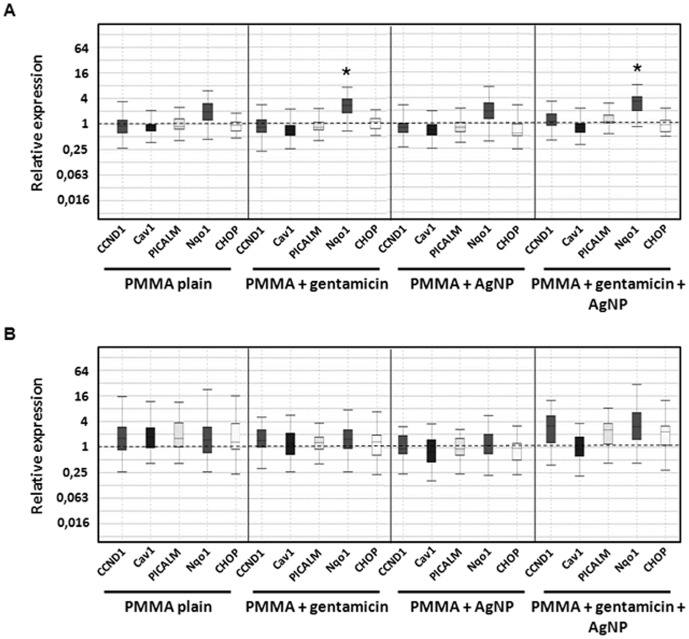
mRNA expression of human mesenchymal stem cells. Cells were incubated with different PMMA bone cements for (A) 24 hours and (B) 21 days. Results were compared to the control without testing material. Data are presented as the boxplot of five individual experiments (*p≤0.05 vs. control w/o testing material).

In MSCs, PMMA loaded with gentamicin and PMMA loaded with gentamicin and AgNP led to a significant induction of the oxidative stress marker Nqo1 after 24 hours. After 21 days the Nqo1 marker for PMMA with gentamicin declined to normal levels, whereas for PMMA with gentamicin and AgNP the marker was still slightly, but not significantly, increased. For the cell stress marker CHOP, that is upregulated during endoplasmatic reticulum stress, no induction caused by the different materials was observed in MSC at any time point. Neither at day 1 nor at day 21 an effect on the proliferation marker CCND1 could be detected. Also no alterations in clathrin- and caveolin-dependent endocytosis, caused by nanoparticle uptake, were observed (Fig. 6).

**Figure 7:**
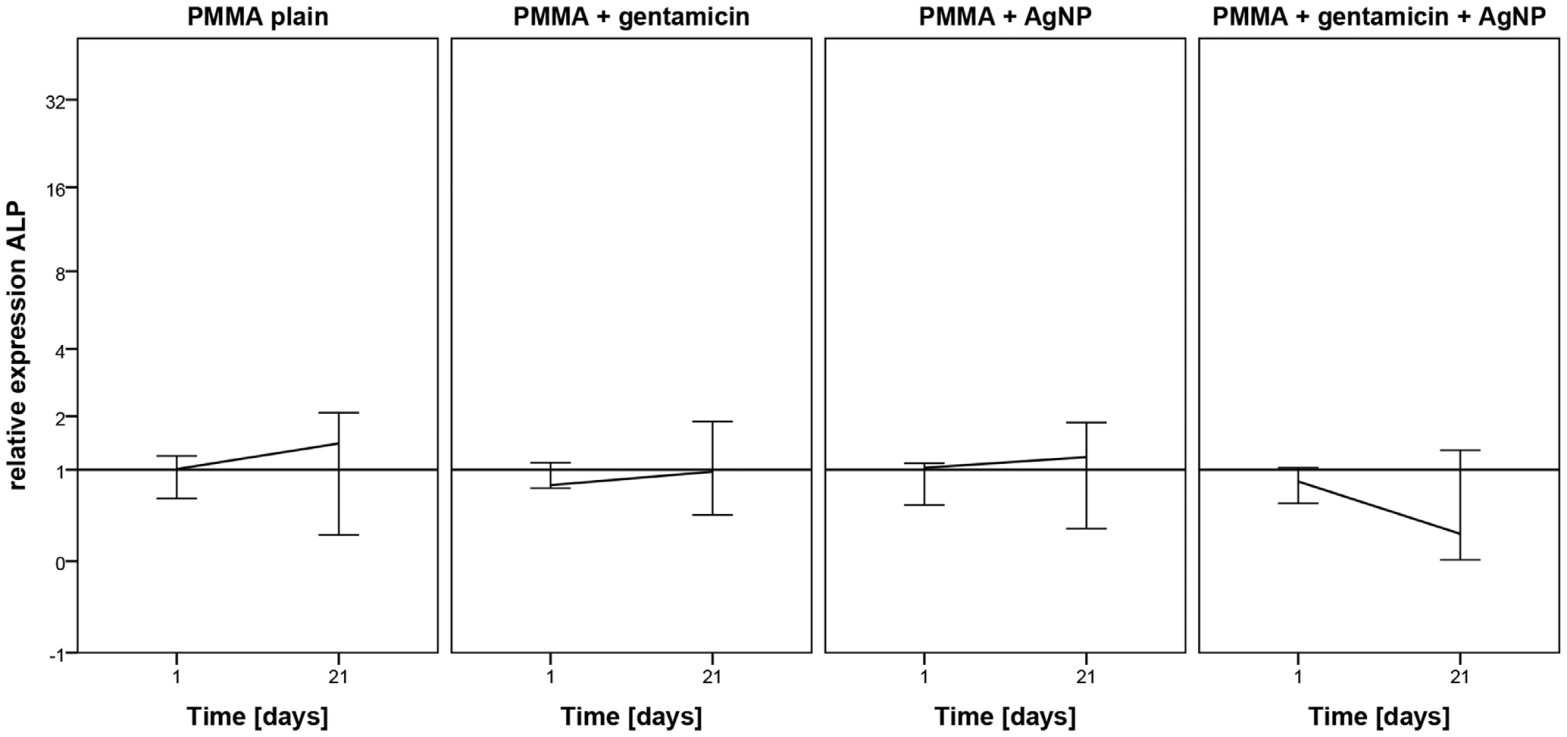
mRNA expression of ALP. Human mesenchymal stem cells were incubated osteogenic differentiation media and with different PMMA bone cements for (A) 24 hours and (B) 21 days. Results were compared to day 1 of control without testing material. Data are presented as the boxplot of five individual experiments.

**Figure 8:**
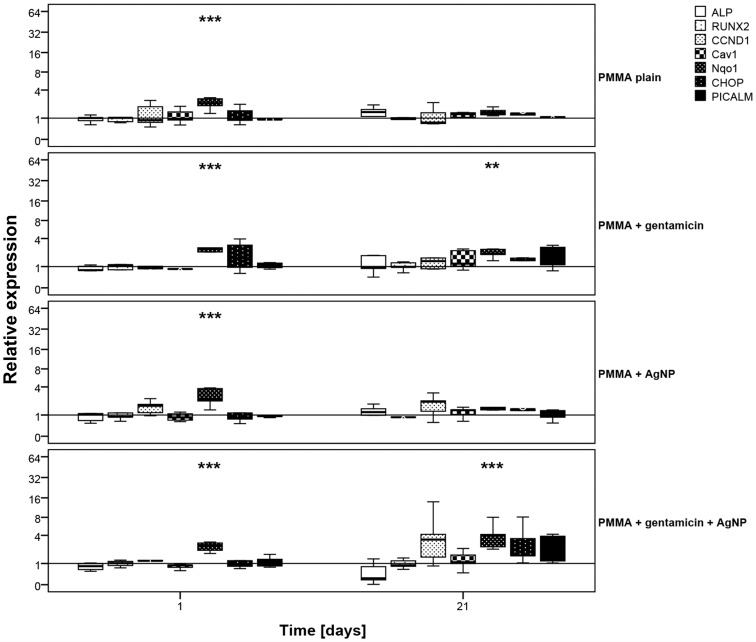
mRNA expression of human mesenchymal stem cell incubated in osteogenic media. Cells were incubated with different PMMA bone cements for (A) 24 hours and (B) 21 days. Results were compared to the control without testing material. Data are presented as the boxplot of five individual experiments (*p≤0.05, **p≤0.01, **p≤0.001 vs. control w/o testing material).

On mRNA level, osteogenic differentiation of MSC-OMs was investigated by the marker ALP for which a slight but not significant increase was detected after incubation with plain PMMA, PMMA loaded with gentamicin, and PMMA loaded with AgNP. A decrease in ALP mRNA expression was found after addition of PMMA loaded with both, gentamicin and AgNP (Fig. 7). For the second tested osteogenic differentiation marker RUNX2 no regulation was determined (Fig. 8).

Also in MSC-OMs, no regulation of the endocytosis markers PICALM and Cav1 caused by the PMMA bone cements was detected. Otherwise, a significant increase in Nqo1 levels, but not in CHOP levels, could be detected in all PMMA groups after 24 hours. After 21 days, only gentamicin loaded PMMA and PMMA supplemented with gentamicin and AgNP still revealed an elevated Nqo1 level (Fig. 8).

## Discussion

PMMA bone cement is a frequently used biomaterial in arthroplasty, cranioplasty and vertebroplasty. To prevent bacteria settlement on the biomaterial surface, PMMA is routinely loaded with antibiotics, like gentamicin or vancomycin. However, despite the prophylactic treatment, the increasing number of multidrug resistant bacteria prevents the efficacy of conventional antibiotics. To assure broad-spectrum antibacterial prophylaxis, including multiresistant bacteria, new antibiotic agents have to be deployed. The excellent antibacterial capacity of AgNP against gram-positive and gram-negative bacteria has been described in several studies. Even eradication of already existing biofilms and inhibition of multidrug resistant pathogens has been demonstrated. Because AgNP target various essential bacterial components simultaneously, there is no risk of developing AgNP-resistance. Contrary to antibiotics incorporated into PMMA, AgNP act directly at the implant surface and have no long-distance effect. Furthermore, AgNP release silver ions over a long period, while release kinetics of antibiotics rapidly decrease after several days, which favors the development of antibiotic-resistant bacteria.

For the successful application of a biomaterial in clinical routine, antibacterial efficacy is not sufficient. It is equally important to ensure acceptable biocompatibility.

In the present study we demonstrated similar viability of MSC for all tested PMMA bone cements as well as for the negative control without material. No decrease in viability was determined. In MSC-OM the highest cell viability was found for plain PMMA and the lowest for PMMA supplemented with gentamicin and AgNP as well as for the negative control without material. Thus, indicating that osteogenic differentiated MSC-OMs are more sensitive to addition of bone implants than original MSCs. Surprisingly, cells incubated with PMMA bone cement showed higher levels of cell viability than the negative controls without material. We suppose that the decrease in viability is resulted by the overcrowded cell culture well after culturing of 10,000 cells per cm^2^ for 21 day. High cell numbers often cause a stop in proliferation, decline viability and subsequently induce apoptosis. However, it is necessary to start cell culture experiment with an appropriate number of cells. Cell number should not be too low because the cells need contact with other cells for stimulation of proliferation and cell growth. On the other side the number of seeded cells should not be too high to allow growth for enhancing the test duration. The here seeded amount of 10,000 cells seems to be a compromise that allows investigations for 21 days and good ongrowth in the wells with PMMA cements. These results agree with the findings of Alt et al., who demonstrated good ongrowth of human osteoblasts on PMMA bone cement loaded with 1% AgNP as well as antibacterial efficacy [32]. Light microscopic observations as shown in Fig. 5 support our hypothesis on the good ongrowth to PMMA bone cements. In addition it shows the high amount of cells in wells without material after 7 days of incubation time. However, the bivalent effect of the amount of seeded cells let us stop the cell culture experiment on day 21. Clinically, bone materials are used for much longer time periods. Therefore it is necessary to perform long time analysis. This is one of the major limitations of the present study. However, in vitro studies with bone implants often use short time cultures [33]–[35] because it is well known that MSC are aging ex vivo. Osteogenic differentiation, proliferation, and viability are decreased in MSCs expanded in long term cultures [36], [37]. In addition several reports describe that the donor age also affects the rate of in vitro senescence in MSC as well as osteogenic differentiation potential of MSC-OMs [38], [39]. Besides the heterogeneity of the used cell populations caused by donor age there is an additional increase in variability supposed that is originated by the alterations in the differentiation potential among single cell clones of MSC [40]. However, since the patients in trauma surgery and orthopedics are also very heterogenic we guess that it is worth analyzing the cytocompatibility for bone implants in primary MSCs and MSC-OM.

The heterogeneity between the donor's cells might also be responsible for the high variance in the mean DNA content in MSC-OM. All PMMA variants as well as cultures without material revealed an increase in DNA content. Usually DNA is increasing when the cells proliferate and the cell number rises. Thus, we suspect that AgNP loaded PMMA as well as PMMA with AgNP and gentamicin does not decline cell number and proliferation potency. From day 7 to day 21 there is an increase in mean DNA content for PMMA with gentamicin reaching levels of plain PMMA. Several release kinetics of gentamicin loaded PMMA have demonstrated drastically decreasing release of gentamicin after a few days [15], [33], [34]. This diminished release of gentamicin sulfate in later time points seems to allow healthy cell growth. Interestingly, there is a disagreement in the literature as to whether gentamicin influences cell proliferation or cell viability [33], [34]. Since the cytotoxicity of gentamicin is dose-dependent [41] we assume that the used PMMA bone cements does not liberate gentamicin concentrations which induce negative effects. In a previous study we demonstrated decreased cell viability as well as cell number caused by AgNP exposure [31]. Cytotoxicity of AgNP on bone cells has also been described by other research groups [35], [42], [43]. The direct contact of the bone cells with the AgNP exposed on the PMMA surface seems to reduce their metabolic activity. This might be due to disruption of mitochondria membrane or mitochondrial function caused by released silver ions as has been described by different research groups [44]–[46]. That there is no decrease of cell number after incubation on AgNP-loaded PMMA can be due to the absence of freely available nanoparticles interfering with DNA replication. However, only a slight increase in DNA content was measured in MSC-OM with and without PMMA bone cements. This might be caused by the addition of osteogenic differentiation media. Usually MSCs stop proliferation and start differentiation if basic growth media is changed into osteogenic differentiation media.

Determination of ALP content using an ALP assay revealed a slight but similar increase in ALP amount from day 1 to 21 for the different PMMA bone cements as for the control without material. To verify this result real time RT PCR was performed using gene specific primers for ALP. A slight but not significant increase in ALP mRNA expression was detected for AgNP loaded, plain, and gentamicin loaded PMMA. PMMA with gentamicin and AgNP showed a decrease in ALP mRNA expression. Thus, loading with both, gentamicin and AgNP, lead to changes in material characteristics that reduce osteogenic differentiation. In addition, due to the altered material characteristics MSC-OMs incubated on PMMA loaded with gentamicin and AgNP arranged in nest-like structures consisting of not fully differentiated cells. In our previous study where we administrated several concentrations of AgNP dispersions on MSCs and MSC-OM we did not determine a reduction in ALP content and mRNA expression [31]. Similar results were described for osteogenic differentiation of human bone marrow-derived MSC and MSC derived from adipose tissue [47], [48]. Using urine-derived stem cells Qin et al. reported an increase in ALP activity after addition of AgNPs whereas murine osteoblasts showed a decrease in ALP activity [35], [49]. It has been also described that application of gentamicin leads to negative effects on ALP activity [41], [50]. Thus, the presented decline in ALP mRNA expression might be due to additive effects of AgNP and gentamicin when both are loaded to PMMA bone cement. Up to our knowledge the combination of gentamicin and AgNPs in PMMA bone cement was not analyzed so far. In wound dressing materials it has already been tested and no significant differences in the healing pattern were observed if AgNP wound dressing was combined with gentamicin [51]. We suppose that the release of gentamicin sulfate from the surface of PMMA loaded with gentamicin and AgNP increases the porosity of the material, thereby enlarging the PMMA surface, which in turn leads to increased liberation of silver ions. As a consequence, there is a synergistic effect of gentamicin and silver toxicity. Further investigations will be necessary to identify a suitable AgNP concentration for combination with gentamicin in PMMA bone cement that will not reveal negative effects.

In our previous study we were also able to link AgNP cytotoxicity to induction of oxidative stress following AgNP uptake [31]. However, in the present work we observed after 24 hours elevated levels of Nqo1 in MSC-OMs for all PMMA variants and in MSCs for PMMA with gentamicin and PMMA with gentamicin and AgNP. After 21 days the Nqo1 levels were also increased for PMMA with gentamicin and AgNP. These results indicate that PMMA itself can induce the production of reactive oxygen species. During the polymerization of methylmethacrylate to PMMA a redox system is used, with benzoyl peroxide (BPO) serving as the initiator and N, N-dimethyl-p-toluidine (DMT) as the accelerator. This system leads to the production of benzoate and free radicals, which in turn can induce oxidative cell stress. Incorporation of radical scavengers, like vitamin E, can improve the cytocompatibility of PMMA [52].

The stress marker CHOP that is upregulated during ER stress, e.g. caused by AgNP-related failure in protein folding [53], was not induced at any time point. Real time RT PCR further revealed no induction in clathrin- or caveolin-dependent endocytosis in all PMMA groups.

Results from cell stress analysis and cellular uptake indicate that AgNP are firmly incorporated into the material and not freely available for endocytosis.

In conclusion, we were able to demonstrate cytocompatibility of AgNP loaded PMMA and PMMA supplemented with gentamicin and AgNP in MSCs and to a slightly lower degree in MSC-OMs in vitro for an observation period of 21 days. Further studies for a longer period of time as well as mechanistic analysis of the induction of cell stress should be performed to predict possible health risks.
